# Atrial Fibrillation Management by Pulmonary Vein Isolation in Patients With Complex Congenital Heart Disease

**DOI:** 10.1016/j.jacasi.2025.12.013

**Published:** 2026-03-03

**Authors:** Masayuki Sakai, Satoshi Higuchi, Yuko Matsui, Shun Hasegawa, Shohei Kataoka, Kyoichiro Yazaki, Kensuke Kikuchi, Shonosuke Watanabe, Kunihiro Kani, Kiyotaka Takefuta, Tetsuri Sakai, Takahiko Kinjo, Keiko Toyohara, Daiji Takeuchi, Hideki Kobayashi, Ayako Okada, Masahiro Yagi, Daigo Yagishita, Morio Shoda, Junichi Yamaguchi

**Affiliations:** a Department of Cardiology, Tokyo Women’s Medical University, Tokyo, Japan; b Clinical Research Division for Heart Rhythm Management, Tokyo Women’s Medical University, Tokyo, Japan; c Department of Pediatric Cardiology, Tokyo Women’s Medical University, Tokyo, Japan; d Department of Cardiovascular Medicine, Shinshu University School of Medicine, Nagano, Japan; e Department of Cardiology, Sendai Cardiovascular Center, Miyagi, Japan

**Keywords:** atrial fibrillation, atrial tachycardia, complex congenital heart disease, pulmonary vein isolation, right atrium

Advances in surgical and medical care have increased survival in congenital heart disease (CHD), resulting in a growing adult population at heightened risk for atrial fibrillation (AF).^1^^,^^2^ Although pulmonary vein isolation (PVI) is an established therapy for AF in structurally normal hearts^3^ and has shown efficacy in atrial septal defect,^4^ its role in complex CHD remains unclear. Unique anatomic substrates—such as surgical scars, conduits, and atrial remodeling—pose additional challenges for ablation. This study aimed to assess the feasibility and outcomes of PVI in patients with complex CHD and to identify specific issues that may guide future strategies.

This multicenter observational study prospectively enrolled consecutive patients with moderate-to-severe CHD undergoing first PVI between 2009 and 2024 at 3 Japanese centers, in accordance with the 2020 European Society of Cardiology guidelines^5^ and the Declaration of Helsinki, with institutional ethics approval (Approval No. 5664) and written informed consent obtained from all participants.

Preprocedural evaluation included echocardiography and multidetector computed tomography, with imaging integrated into electroanatomical mapping systems. Anticoagulation was standardized, and transesophageal echocardiography was performed when indicated to exclude left atrial (LA) thrombus. Procedures were performed under deep sedation with heparin anticoagulation (target activated clotting time 300-350 seconds). Following transseptal puncture, 3-dimensional mapping (CARTO, Biosense Webster)–guided radiofrequency ablation (25-50 W). Circumferential PVI was the primary endpoint, confirmed by entrance/exit block. Empirical superior vena cava (SVC) isolation was performed unless no potentials were present. Concomitant atrial tachycardia (AT) ablation was performed using activation/entrainment mapping, with lesion sets tailored to scar-related or macro–re-entrant mechanisms. No routine LA substrate modification was undertaken.

Patients were followed at 1, 3, 6, 9, and 12 months, then biannually. Recurrence was defined as atrial tachyarrhythmias (ATAs) >30 seconds beyond a 3-month blanking period. Repeat ablations focused on reisolating reconnected PVs or SVC, with additional AT ablation as per the initial strategy.

Continuous variables are presented as mean ± SD or median (IQR). Group comparisons used appropriate parametric or nonparametric tests. Freedom from ATA and AF recurrence was analyzed using Kaplan-Meier methods with log-rank testing. A 2-sided *P <* 0.05 was considered statistically significant.

A total of 32 patients with complex CHD underwent their first PVI. Among them, 24 (75.0%) had moderate CHD—most commonly repaired tetralogy of Fallot (n = 12, 37.5%)—and 8 (25.0%) had severe CHD, with corrected transposition of the great arteries being the most frequent (n = 4, 12.5%) (Figure 1A). Prior cardiac surgery had been performed in 25 (78.1%), all involving right atrial incisions. The mean age at ablation was 55.7 ± 13.4 years (moderate: 58.6 ± 13.8 years; severe: 47.0 ± 11.0 years), and 24 (75.0%) were men (moderate: 17 of 24, 70.8%; severe: 7 of 8, 87.5%). Paroxysmal AF was present in 18 (56.3%), including 14 of 24 (58.3%) with moderate CHD and 4 of 8 (50.0%) with severe CHD. Median LA volume index was 34.5 mL/m^2^ (Q1-Q3: 18.1-45.5 mL/m^2^) (moderate: 33.1 mL/m^2^ [Q1-Q3: 22.1-42.4 mL/m^2^]; severe: 48.4 mL/m^2^ [Q1-Q3: 25.7-77.7 mL/m^2^]). Systemic ventricular ejection fraction averaged 53.0 ± 16.3% (moderate: 51.0% ± 15.2%; severe: 63.2% ± 17.7%). Twelve patients (37.5%) had undergone prior AT ablation, including 8 (25.0%) with prior cavotricuspid isthmus (CTI) ablation.

**Figure 1:**
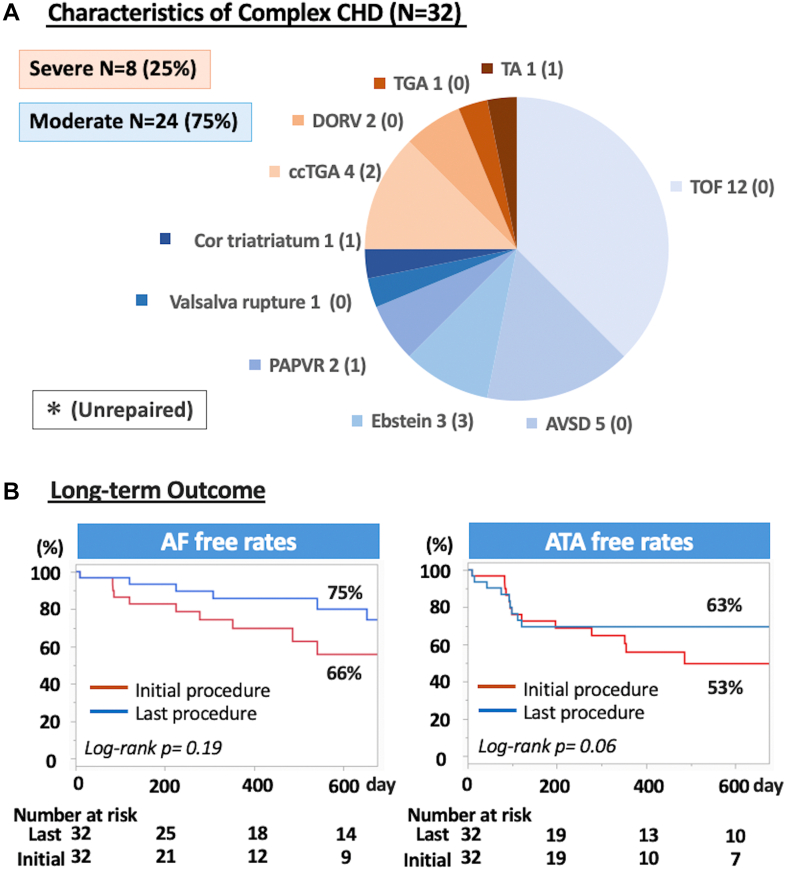
Pulmonary Vein Isolation for AF in Complex CHD (A) Types of moderate-to-severe congenital heart disease (CHD). (B) Kaplan-Meier curves showing freedom from atrial fibrillation (AF) (left) and any atrial tachyarrhythmia (ATA) (right). Red curves represent the initial procedure, and blue curves represent repeat procedures. AT = atrial tachycardia; AVSD = atrioventricular septal defect; ccTGA = congenitally corrected transposition of the great arteries; DORV = double-outlet right ventricle; PAPVR = partial anomalous pulmonary venous return; TA = tricuspid atresia; TGA = transposition of the great arteries; TOF = tetralogy of Fallot.

All patients underwent successful PVI. At baseline, 14 of 32 patients (43.8%) were in AF; AF terminated during PVI in 3 (9.4%), while the remaining required cardioversion. Pulmonary vein automaticity was observed in 20 of 32 (62.5%), and SVC isolation was performed in 12 (37.5%). Concomitant AT occurred in 15 of 32 (46.9%), all originating from the RA (moderate: 12 of 24, 50.0%; severe: 3 of 8, 37.5%). LA AT was rare (2 of 32, 6.3%). CTI-dependent atrial flutter was the most frequent AT (12 of 32, 37.5%), followed by RA scar-related AT (3 of 32, 9.4%). Median procedure time was 215 minutes (Q1-Q3: 154-312 minutes), longer in severe than moderate CHD (316 minutes [Q1-Q3: 245-386 minutes] vs 200 minutes [Q1-Q3: 153-250 minutes]).

During a median follow-up of 22 months (Q1-Q3: 11-42 months), 17 of 32 (53.1%, 95% CI: 34.7%-70.9%) remained free from any ATA recurrence, and 21 of 32 (65.6%, 95% CI: 46.8%-81.4%) were free from AF recurrence (Figure 1B). Recurrent arrhythmias included AF only in 7 (21.9%), AT only in 5 (15.6%), and both AF and AT in 4 (12.5%).

A second ablation was performed in 8 of 32 patients (25.0%) at a median of 10.7 months (Q1-Q3: 6.6-13.4 months). During repeat procedures, AT was inducible in 6 of 8 (75.0%), predominantly of RA origin, including RA scar-related AT (n = 3, 37.5%) and CTI-dependent flutter (n = 3, 37.5%). PV reconnection was observed in 5 of 8 (62.5%), and SVC reconnection in 1 of 8 (12.5%). Following a total of 46 procedures, freedom from ATA recurrence improved (log-rank *P =* 0.06; 95% CI: 43.7%-78.9%), with AF-specific freedom also improved (log-rank *P =* 0.19, 95% CI: 56.6%-88.5%) (Figure 1B).

Periprocedural complications occurred in 4 of 46 procedures (8.7%), including pericardial effusion, hemothorax, ischemic stroke, and coronary vasospasm; all were managed conservatively without procedure-related deaths. Importantly, heart failure hospitalization decreased from 12 of 32 (37.5%) in the year before ablation to 2 of 32 (6.3%) in the first year postablation (*P <* 0.005). No deaths occurred during follow-up.

AF in complex CHD carries high risks of heart failure, thromboembolism, and mortality, making rhythm control a key therapeutic goal. Our findings highlight 3 main points. First, PVI alone suppressed AF in a subset of patients, indicating a potential contribution of PV triggers even in complex CHD. Second, recurrent arrhythmias were predominantly right atrial ATs, underscoring the limitations of PVI alone and the importance of adjunctive AT ablation. Third, after multiple procedures, effective control of both AF and AT was associated with a marked reduction in heart failure hospitalizations.

Unlike prior studies that included patients with mild CHD or heterogeneous ablation strategies, we exclusively investigated moderate-to-severe CHD using a uniform thoracic vein–centered approach. Nevertheless, the marked heterogeneity in hemodynamics across CHD subtypes suggests that PVI alone is unlikely to be universally sufficient. Importantly, concomitant AT was highly prevalent in this population: 37.5% of patients had undergone prior right-sided AT interventions before AF diagnosis, and AT was present or inducible in the majority. These findings help explain why a strategy that combined PVI with additional AT ablation proved highly effective, even when repeat procedures were required. Finally, although the sample size was limited, our results raise the possibility that a rhythm control approach centered on thoracic vein isolation may help reduce heart failure hospitalizations—an outcome of critical importance given the burden of heart failure in this population.

Several limitations should be noted. The cohort was small and heterogeneous, Fontan and atrial switch patients were excluded, and arrhythmia recurrence may have been underestimated because of variable follow-up. The diverse anatomy of adult CHD also limits the universal applicability of PVI alone. In addition, the long enrollment period may have influenced outcomes because of evolving ablation technologies. Larger prospective studies are needed to validate these findings.

In conclusion, PVI may play a role in managing AF in patients with complex CHD. In some cases, PVI helped reduce AF recurrence, and when combined with concomitant AT ablation, it was associated with fewer heart failure hospitalizations related to ATA. However, the frequent coexistence of AT underscores the need for additional substrate modification, and further prospective studies are warranted.

## Funding Support and Author Disclosures

Drs Higuchi, Yagishita, and Shoda belong to the same endowed department established by contributions from Medtronic Japan, Boston Scientific, Biotronik Japan, and Abbott Medical. All other authors have reported that they have no relationships relevant to the contents of this paper to disclose.
